# Utilizing the focused conversation method in qualitative public health research: a team-based approach

**DOI:** 10.1186/s12913-019-4107-0

**Published:** 2019-05-14

**Authors:** Charissa Fritzen-Pedicini, Susan C. Bleasdale, Lisa M. Brosseau, Donna Moritz, Monica Sikka, Emily Stiehl, Rachael M. Jones, for the CDC Prevention Epicenter Program

**Affiliations:** 1 Division of Environmental and Occupational Health Sciences, School of Public Health, University of Illinois at Chicago, 1603 W Taylor Street (M/C 923), Chicago, IL 60612 USA; 2 Division of Infectious Diseases, College of Medicine, University of Illinois at Chicago, Chicago, USA; 3 Division of Health Policy and Administration, School of Public Health, University of Illinois at Chicago, Chicago, USA

**Keywords:** Focused conversation method, Interdisciplinary, Qualitative data analysis, Semi-structured interviews, Team science

## Abstract

**Background:**

Qualitative research studies are becoming increasingly necessary to understand the complex challenges in the healthcare setting. Successfully integrating interdisciplinary teams of investigators can be challenging, as investigators inherently view data through their disciplinary lens. Thus, new methods, such as focused conservation, are needed to facilitate qualitative data analysis by interdisciplinary teams. The purpose of this manuscript is to provide a clear description of how we implemented the focused conversation method to facilitate an organized data-driven discussion that responded to our study objectives and ensured participation of our interdisciplinary team. The focused conversation method has not, to our knowledge, been utilized for this purpose to date.

**Methods:**

To better understand the experience of healthcare personnel (HCP) during preparations for the 2014–2015 Ebola Virus Disease (EVD) outbreak, we interviewed HCP who participated in decision making about EVD preparations and training of workers in the use of enhanced personal protective equipment ensembles in the metropolitan Chicagoland area of Illinois to attain a priori research objectives. We identified a systematic method – the focused conversation method – that enabled our interdisciplinary team to interactively contribute to the framing, analysis and interpretation of the data that would enable us to focus on our research objectives.

**Results:**

The focused conversation developed to support our a priori research objective about the training of HCP in preparations included objective, reflective, interpretive and decisional questions. These questions grounded the conversation in the data, while leveraging discipline-specific lenses and professional experience in the analysis and interpretation. Insights from the conversation were reviewed later against interview transcripts to ensure validity. The conversation identified areas for future research directions and deficiencies in the interview instrument.

**Conclusions:**

The focused conversation is an efficient, organized method for analysis of qualitative data by an interdisciplinary team.

## Background

Incorporating qualitative methods into infection control and other applied health research has been increasing in recent years because these methods can add richness and explanation to complex systems in a way that cannot be described through quantitative variables [1, 2]. Moreover, interdisciplinary studies are becoming increasingly common in this research area, involving research teams with multiple disciplines represented, including: qualitative and quantitative researchers, healthcare personnel, epidemiologists, occupational health, and infection control professionals, and others [3]. Results from qualitative studies utilizing interdisciplinary research teams can provide insight into decision-making processes, institutional management and culture, and systems challenges [3, 4]. Qualitative research methodology is diverse, with approaches stemming from multiple social sciences, including psychology, sociology and anthropology, as in ethnography and phenomenology [5, 6]. Grounded theory, a common methodical approach in qualitative research, develops a theory inductively based on analysis of data [7]. Each qualitative method has strengths and limitations, and must be chosen with careful consideration of study aims and objectives.

As part of a study about how acute care hospitals in Chicago, Illinois prepared for the 2014–2015 Ebola Virus Disease (EVD) we performed semi-structured interviews with healthcare personnel (HCP) who participated in decision making about EVD preparations or training of workers in the use of enhanced personal protective equipment. The objectives of these interviews were to understand: i) how personal protective equipment (PPE) ensembles, used to protect HCP from infection, were selected, ii) considerations for PPE acquisition and use, and iii) how training in the use of PPE ensembles and EVD patient care was developed and delivered. In planning this research, we conducted a literature review and consulted with colleagues with expertise in qualitative research methods, but found many of the methods difficult to apply in our context owing to our need to evaluate a priori research objectives and involve our team of interdisciplinary investigators. Though we were interested in emerging themes, a priori objectives were prioritized based on the norms of our disciplines and the extent of existing knowledge about the experience of hospitals preparing for EVD. Our core team of investigators included: three infectious disease physicians, two of whom have roles in infection prevention; two industrial hygienists, a specialty within occupational health, with expertise in personal protective equipment; and a public health professional with experience in infectious diseases. We wanted all team members to engage equally, bringing their experiences and disciplinary training to bear on a complex problem. We identified the focused conversation method to be capable of enabling analysis of the interview data, while achieving our a priori research objectives and engaging our team.

The focused conversation method involves answering, through conversation, a series of questions organized into four phases (objective, reflective, interpretative and decisional) that draw upon ways in which humans process and make judgments about information, while promoting order and systematic dialogue [8]. The focused conversation method has traditionally been used in business and education to design and guide conversations so as to ensure that the goal of the conversation is achieved [9]. The conversation involves stakeholders and other individuals interested in the outcome, and should be predicated by a review of relevant data/information by participants and setting of a goal, which informs the development of questions. For example, one author (RMJ), has used the focused conversation method in faculty meetings about curriculum revision, where data have included existing courses, course learning objectives, program competencies, and requirements from accreditors, and questions were written to lead to decisions about shared competencies across programs and the design of new courses. The focused conversation method is beginning to be used in health sciences research. For example, Caress, et al. [10] used focused conversation style interviews to collect data, while Realpe, et al. [11] employed the focused conversation method in combination with thematic analysis to analyze conversations from patient recruitment and diagnostic consultations. Details of how and why the focused conversation method was employed in these studies, however, were not well described.

With the goal of enhancing the use of the focused conversation method, we wanted to share our approach for using this method for qualitative data analysis. The objective of this manuscript is to clearly describe how we implemented the focused conversation method to facilitate analysis of data that responded to a priori research objectives and ensured participation of our interdisciplinary team. Ultimately, we employed the focused conversation method twice in this study to separately analyze data related to study objectives about the selection and use of PPE and about training, though this work centers on the focused conversation about training. The results are presented elsewhere (Bleasdale SC, Sikka MK, Brosseau LM, Fritzen-Pedicini C, Moritz D, Stiehl E, Jones RM: Experience of Chicagoland acute care hospitals in preparing for Ebola Virus Disease, 2014–2015, Submitted). Further, we also propose a number of practical suggestions for other researchers who want to apply the focus conversation method in future research.

## Methods

### Research team

Our research team was diverse with respect to disciplinary training and experience. The team included two industrial hygienists, three infectious disease physicians and a public health professional. The public health professional and one physician were relatively junior, within 3–5 years of their academic training, and the remaining investigators were mid-career and senior faculty.

### Interviews

A total of 28 interviews, conducted with participants from 15 acute care hospitals, were analyzed. Purposeful sampling was used to identify hospitals for recruitment, and contact was first initiated through infection prevention or emergency management offices. Broadly, the interview addressed four themes: participant characteristics; institution organization and culture; training experiences; and experience with PPE. The participant recruitment strategy, participants and interview instrument are described in more detail elsewhere (Bleasdale SC, Sikka MK, Brosseau LM, Fritzen-Pedicini C, Moritz D, Stiehl E, Jones RM: Experience of Chicagoland acute care hospitals in preparing for Ebola Virus Disease, 2014–2015, Submitted).

### Interview analysis: codebook development and coding

An iterative approach was used for codebook development. In each iteration, multiple investigators coded a subset of interviews and then discussed the experience. Ambiguity in the code interpretation was reduced by revising codes, adding definitions and adding examples for each code. Ultimately, the code book included 15 parent codes: 1) interviewee role; 2) management response; 3) changing dynamics; 4) external agencies; 5) trainer characteristics; 6) trainee characteristics; 7) training; 8) suspect EVD patients; 9) operational disruptions; 10) PPE; 11) challenges, 12) lessons learned and comparisons; 13) emotions and perceptions; 14) support for employees and occupational health; 15) ethical issues; and 16) media. Two investigators coded each interview transcript, and a third investigator applied a series of rules to reconcile the codes of the two investigators. Assignment of coders was purposeful, to ensure different combinations of investigators coded transcripts and reconciled codes. The rules for reconciliation were: i) no new codes can be added to the text, ii) if two coders had matching codes, those codes must be included in final code decision, and iii) if there are more than five relevant codes for the text, collapse sub-codes into the five most relevant parent codes. This approach was used to capture and manage variation in coding that could result from the investigators’ disciplinary perspectives and experience. The final codebook is available elsewhere (Bleasdale SC, Sikka MK, Brosseau LM, Fritzen-Pedicini C, Moritz D, Stiehl E, Jones RM: Experience of Chicagoland acute care hospitals in preparing for Ebola Virus Disease, 2014–2015, Submitted).

### Data analysis and interpretation: the focused conversation

The focused conversation method involves a series of questions organized to draw upon ways in which humans process and make judgments about information while promoting order and systematic dialogue [9]. Specifically, questions are organized into four phases:

1. The *objective phase* includes questions asking what research team members think the data show, what appears to be known and what appears to be left unknown.
2. The *reflective phase* includes questions that ask research team members to reflect on their response, on other team members’ responses, and on knowns and unknowns.
3. The *interpretative phase* includes questions that push research team members to consider the meaning of the data and their responses to it.
4. The *decisional phase* includes questions that ask how research team members can move forward towards their objectives and goals, often including plans for future action.

## Results

The focused conversation method was implemented as follows. Investigators developed a focused conversation guide with consideration for the a priori study objectives after the interview transcripts had been coded. Parent codes and sub-codes relevant to each study objective were identified, and the sections of interview transcripts to which these codes had been assigned were extracted from the dataset. For the training-related focused conversation, interview text assigned parent codes 5–7 were extracted. (For the PPE-related focused conversation, interview text assigned parent codes 2 and 10 were extracted.) Each investigator was given the objective questions from the focused conversation guide and individually spent 3–10 h identifying the data (sections of interview transcripts) that addressed those questions. This was done to ensure that all investigators read all of the relevant data and to set the frame for the conversation.

After this initial data review, investigators met and discussed all of the questions in the focused conversation guide. Table 1 displays the focused conversation guide developed for the study objectives about training during EVD preparations. A separate focused conversation guide was developed for the study objectives about the selection of PPE. The focused conversation was audio recorded and notes were taken to serve as a written record of the team data analysis and interpretation. The focused conversation occurred over 2–3 h.

**Table 1:** Focused conversation used for training related research objective

Focused Conversation Phase	Purpose of Phase	Questions	Question Rationale
Objective	Ensures all objective details are reviewed by research team prior to data interpretation. Sets up the framework for the focused conversation, identifies that data that we want to focus on in relation to our study aims. Focus on the objective data without making judgements or inferences about the participant responses	1. How was training delivered?	Understand what institutions did during the 2014–2015 outbreak of EVD with respect to training about the use of PPE or the care of EVD patients.
		2. Who developed training?	
		3. Who delivered training?	
		4. How was the effectiveness of training evaluated?	
		5. What was the content of training for Ebola patient care (topics and skills)?	Understand how institutions supported and defined the scope of training.
		6. What technology was used for training, if any?	
		7. How were skills maintained over time?	
		8. What institutional support was provided for training?	
Reflective	Allows team members to express and discuss how the data related to our professional training, content knowledge and experiences.	1. Were the appropriate topics and skills addressed in training?	Explore our research team’s response to the data in relation to training content and modality, including contrasting our expectations and values with the experiences of participants
		2. What topics and/or skills do you think should be included in training?	
		3. What aspects of training appeared to be positively received by trainees or by the institution?	
		4. Were you surprised by the responses? Why?	
Interpretive	Guides research team to address the meaning of the data as it relates to our study aims and objectives. Guides research team to integrate the data into a shared overall framework.	1. How did knowledge or risk perceptions influence the design of training for Ebola?	Based on our interpretation of the data, determine how participant experiences can inform future training for infection prevention. Based on our interpretation of the data, identify challenges in training design and implementation.
		2. How did healthcare personnel’s knowledge affect training participation and compliance?	
		3. What were barriers to training? Consider the institution and individuals.	
		4. What did the Ebola training experience say about training for routine infection prevention?	
		5. What does this tell us about Ebola preparedness/training in Chicagoland healthcare facilities?	
Decisional	Encourages team members to come to consensus on implication of the data in relation to larger aims and goals. Encourages team members to extend their theoretical findings to practical or policy actions.	1. What types of training should be used going forward to prepare for emerging diseases/high consequence infections?	Based on our interpretation of the data and conversation, identify factors that should be considered when designing and implementing training of emerging infectious diseases and high-consequence infections in the future? Based on our interpretation of the data and conversation, identify measures for determining staff and institution preparedness.
		2. What are the characteristics to consider when creating training for PPE use for emerging diseases/high-consequence infections?	
		3. Training is designed to alter worker behavior; what must an institution prepare to ensure workers can employ the desired behavior?	
		4. What are good measures for determining worker and institutional preparedness for emerging diseases/high-consequence infections?	

As a group we first discussed the objective questions, which allowed all team members to get an overview of the data obtained from the interviews – e.g., who developed and delivered training, and the training and evaluation methods used (Table 1). During this discussion we determined it would be beneficial to create a table of indicator and factor variables for validation of our results and interpretation. These indicator and factor variables tabulated specific pieces of information reported by participants, such as: the job roles of trainees, use of in-person practice-based training for PPE, and use of simulated body fluid to evaluate self-contamination during doffing of PPE.

The reflective questions allowed investigators to express any feelings or reactions that were elicited during data review. This phase was an opportunity for investigators to share ways in which the data related to their own professional experiences. Given our diverse professional backgrounds this was useful for allowing team members to understand others’ points of reference. We discussed our perceptions of whether training modality and content seemed appropriate and identified what seemed to be “universal” issues amongst the participants. During this phase we identified that we couldn’t answer all of the questions in the focused conversation guide, such as questions around trainee responses to training. This reflected a disconnect between the interview instrument and our research goals, though we had used a survey to capture trainee perceptions and experiences with training (Bleasdale SC, Sikka MK, Brosseau LM, Fritzen-Pedicini C, Moritz D, Stiehl E, Jones RM: Experience of Chicagoland acute care hospitals in preparing for Ebola Virus Disease, 2014–2015, Submitted).

The interpretive questions allowed the investigators to discuss the challenges and successes of the participants’ experiences and how their experiences could change preparedness strategies for future emerging infectious diseases and/or high consequence infections. During the discussion, we found that some of our interpretive questions could not be answered based on our participant responses, potentially due to limitations in our interview instrument design. This inspired us to identify topics that should be investigated in future research such as: determining rationale for choosing training modalities and exploring experiences in collaborations between healthcare facilities and other public health agencies.

The decisional questions guided our team in summarizing the participants’ overall experiences with respect to developing and delivering training, and address the study objectives. Specifically, we interpreted the participants’ experiences to propose strategies that would better prepare hospitals for future outbreaks of high-consequence infectious, such as support for choosing appropriate PPE and incorporating hands-on training in the use of PPE into training for new employees (Bleasdale SC, Sikka MK, Brosseau LM, Fritzen-Pedicini C, Moritz D, Stiehl E, Jones RM: Experience of Chicagoland acute care hospitals in preparing for Ebola Virus Disease, 2014–2015, Submitted). This phase allowed us to evaluate attainment of the study objective.

### Data validation

After the focused conversation, two investigators individually revisited the data to perform two tasks. The first task was to summarize and organize the data relating to our objective questions. The table included references to participant responses in interview transcripts as a means of validation. Extracting and organizing this data into a table served as a useful reference document and allowed us to visually recognize new patterns and confirm the patterns we discussed in the workshop. It also allowed for another opportunity to meet in small groups and discuss specific participant responses to ensure we were interpreting the data consistently between investigators.

The second task was to identify quotes that supported and challenged our interpretations from the interpretive and decisional phases of our workshop discussion. This provided another opportunity to reexamine the data using comparisons. We used two types of comparisons: i) comparison between interviews of the same group – comparing responses from participants who worked at the same facility and responses from participants who worked at the same classification of facility, and ii) comparisons of interviews of different groups – comparing responses of participants from different facility classifications [12]. This allowed us to explore similarities and differences between interviewees, job titles, and institutions. Investigators kept notes during this secondary data analysis.

The use of the focused conversation method, followed by re-visiting and organizing participant quotes to support and challenge our findings, was an effective means of data validation. Given the standardization of this method, we believe our findings would be reproducible.

## Discussion

Overall, our research team enjoyed engaging with the data and with each other through the focused conversation method, as described. Qualitative research is not the dominant research approach in either industrial hygiene or infectious disease medicine, and the structure of the focused conversation method increased accessibility of qualitative data analysis for research team members with less experience in these methods. Further, the use of multiple validation approaches, particularly tabulating indicator and factor variables increased comfort with and confidence in the focused conversation outcomes.

Within the framework of team science [13], our group of investigators would be defined as a science team engaged in interdisciplinary research that integrates perspectives of multiple disciplines to solve problems related to preparations for emerging, high-consequence infectious diseases in acute care hospitals. Preparations for the 2014–2015 EVD outbreak encompassed most departments of healthcare facilities and many types of professionals [1, 14], thus it was essential that our team include experts from multiple disciplines relevant to protecting workers and patients from high-consequence infectious diseases. Primary team members had expertise in industrial hygiene (RMJ, LMB), infectious disease medicine (SCB, MS, DM) and public health (CFP), and were aided by a colleague with expertise in qualitative research methods and knowledge of occupational health and organizational behavior (ES). We perceive the diversity of the research team to be a strength of this research [15].

The implementation of team science has many challenges, including [13, 16, 17]: divergent philosophies and styles arising from disciplinary expectations and individuality; time and effort to maintain communication; and defining roles and responsibilities. Our team experienced these challenges, but was also challenged to identify a research method that enabled participation by all members in the analysis of data obtained through semi-structured interviews, and accommodated our need to attain a priori research objectives: We found that the focused conversation method fulfilled these needs. Hesse-Biber [18] described how the interdisciplinary research framework challenges disciplinary categories of analysis, which can move teams towards a practical, pragmatic methodologic approach that avoids difficult epistemological or philosophical issues. We found the focused conversation method to be highly practical and pragmatic, though the organization of the questions prompted the investigators to identify and challenge their disciplinary paradigms while remaining focused on the very concrete problem of infectious disease preparedness. However, limitations of disciplinary methodologies in truly interdisciplinary or transdisciplinary research should be more formally described, and new methods that reflect the demands of interdisciplinary or transdisciplinary research developed. This remains a gap in team science research, which has been primarily focused on team formation and dynamics, rather than how team science research is performed [16].

We valued that the focused conversation method enabled an organized, targeted conversation among our interdisciplinary team that was grounded in data and addressed our a priori research objectives, while also eliciting disciplinary paradigms and personal experiences. A particular strength of the method in the context of team science was that it enabled equal participation from all members, regardless of seniority and disciplinary training. Mentorship of junior colleagues has been identified as a value of team science [16], and our implementation of the focused conversation engaged junior colleagues as valued partners. Team members enjoyed the opportunity to engage in scientific conversation during the focused conversations, and such experiences contribute to building trust and inter-personal relationships which are important to maintaining effective research teams [19]. Based on our experience, we suggest that others consider this method for analysis of qualitative data, such as obtained by semi-structured interviews, particularly in the context of interdisciplinary or transdisciplinary research.

## Conclusions

The focused conversation method supported analysis that utilized the interdisciplinary training of our research team to achieve our research objectives. A strength of the approach was that the conversation design led to the evaluation of the study objectives, and to identification of research questions for future work. During the conversation, we identified gaps in the data, suggesting limitations of the interview instrument. The focused conversation method fits well within the framework of interdisciplinary team science. Future work should extend the description of our experience towards development of a user-guide.

## Abbreviations

EVD: Ebola Virus Disease
HCP: Healthcare personnel
PPE: Personal protective equipment

## References

[1] Forman J, Creswell JW, Damschroder L, Kowalski CP, Krein SL (2008). Qualitative research methods: key features and insights gained from use in infection prevention research. Am J Infect Control.

[2] Patton MQ (2002). Qualitative research and evaluation methods.

[3] Gale NK, Heath G, Cameron E, Rashid S, Redwood S (2013). Using the framework method for the analysis of qualitative data in multi-disciplinary health research. BMC Med Res Methodol.

[4] Fitzpatrick R, Boulton M (1994). Qualitative methods for assessing health care. Qual Health Care.

[5] Denzin NK, Lincoln YS (2011). The sage handbook of qualitative research, 4^th^ Edition.

[6] Ritchie J, Lewis J, Nicholls CM, Ormston R (2013). Qualitative research practice: a guide for social science students and researchers, 2^nd^ Edition.

[7] Glaser BG, Strauss AL, Strutzel E (1968). The discovery of grounded theory; strategies for qualitative research. Nurs Res.

[8] Stanfield B, Canadian Institute of Cultural Affairs (2013). The art of focused conversation: 100 ways to access group wisdom in the workplace.

[9] Spee JC (2005). Using focused conversation in the classroom. J Manage Ed.

[10] Caress A-L, Luker K, Woodcock A, Beaver K (2002). An exploratory study of priority information needs in adult asthma patients. Patient Ed Counsl.

[11] Realpe A, Adams A, Wall P, Griffin D, Donovan JL (2016). A new simple six-step model to promote recruitment to RCTs was developed and successfully implemented. J Clin Epidemiol.

[12] Boeije HA (2002). Purposeful approach to the constant comparative method in the analysis of qualitative interviews. Qual Quant.

[13] National Research Council. Enhancing the Effectiveness of Team Science. Washington D.C.: The National Academies Press. 10.17226/19007 26247083

[14] Leonhardt KK, Keuler M, Safdar N, Hunter P (2016). Ebola preparedness planning and collaboration by two health Systems in Wisconsin, September to December 2014. Disaster Med Public Health Prep.

[15] Conlon C, Carney G, Timonen V, Scharf T (2015). “Emergent reconstruction” in grounded theory: learning from team-based interview research. Qual Res.

[16] DeHart D (2017). Team science: a qualitative study of benefits, challenges and lessons learned. Soc Sci J.

[17] Stokols D, Shalini M, Moser RP, Hall KL, Taylor BK (2008). The ecology of team science: understanding contextual influences on transdisciplinary collaboration. Am J Prev Med.

[18] Hesse-Biber S (2016). Doing interdisciplinary mixed methods healthcare research: working the boundaries, tensions, and synergistic potential of team-based research. Qual Health Res.

[19] Bennett LM, Gadlin H (2012). Collaboration and team science: from theory to practice. J Investig Med.

